# Lymphatic Endothelial-to-Myofibroblast Transition: A Potential New Mechanism Underlying Skin Fibrosis in Systemic Sclerosis

**DOI:** 10.3390/cells12172195

**Published:** 2023-09-01

**Authors:** Irene Rosa, Eloisa Romano, Bianca Saveria Fioretto, Khadija El Aoufy, Silvia Bellando-Randone, Marco Matucci-Cerinic, Mirko Manetti

**Affiliations:** 1 Section of Anatomy and Histology, Department of Experimental and Clinical Medicine, University of Florence, 50134 Florence, Italy; irene.rosa@unifi.it (I.R.); biancasaveria.fioretto@unifi.it (B.S.F.); 2 Section of Internal Medicine, Department of Experimental and Clinical Medicine, University of Florence, 50134 Florence, Italy; khadija.elaoufy@unifi.it (K.E.A.); silvia.bellandorandone@unifi.it (S.B.-R.); 3 Division of Rheumatology, Azienda Ospedaliero-Universitaria Careggi (AOUC), 50141 Florence, Italy; 4 Unit of Immunology, Rheumatology, Allergy and Rare Diseases (UnIRAR), IRCCS San Raffaele Hospital, 20132 Milan, Italy; matuccicerinic.marco@hsr.it; 5 Imaging Platform, Department of Experimental and Clinical Medicine, University of Florence, 50134 Florence, Italy

**Keywords:** systemic sclerosis, lymphatic endothelial cells, endothelial-to-myofibroblast transition, myofibroblasts, skin, fibrosis

## Abstract

At present, only a few reports have addressed the possible contribution of the lymphatic vascular system to the pathogenesis of systemic sclerosis (SSc). Based on the evidence that blood vascular endothelial cells can undertake the endothelial-to-myofibroblast transition (EndMT) contributing to SSc-related skin fibrosis, we herein investigated whether the lymphatic endothelium might represent an additional source of profibrotic myofibroblasts through a lymphatic EndMT (Ly-EndMT) process. Skin sections from patients with SSc and healthy donors were immunostained for the lymphatic endothelial cell-specific marker lymphatic vessel endothelial hyaluronan receptor-1 (LYVE-1) in combination with α-smooth muscle actin (α-SMA) as the main marker of myofibroblasts. Commercial human adult dermal lymphatic microvascular endothelial cells (HdLy-MVECs) were challenged with recombinant human transforming growth factor-β1 (TGFβ1) or serum from SSc patients and healthy donors. The expression of lymphatic endothelial cell/myofibroblast markers was measured by quantitative real-time PCR, Western blotting and immunofluorescence. Collagen gel contraction assay was performed to assess myofibroblast-like cell contractile ability. Lymphatic endothelial cells in intermediate stages of the Ly-EndMT process (i.e., coexpressing LYVE-1 and α-SMA) were found exclusively in the fibrotic skin of SSc patients. The culturing of HdLy-MVECs with SSc serum or profibrotic TGFβ1 led to the acquisition of a myofibroblast-like morphofunctional phenotype, as well as the downregulation of lymphatic endothelial cell-specific markers and the parallel upregulation of myofibroblast markers. In SSc, the Ly-EndMT might represent a previously overlooked pathogenetic process bridging peripheral microlymphatic dysfunction and skin fibrosis development.

## 1. Introduction

Systemic sclerosis (SSc, also known as scleroderma) is a complex and orphan connective tissue disorder characterized by multisystem clinical symptoms with a variable and unpredictable course [1,2,3]. Although the exact pathogenetic mechanisms have yet to be clarified, it is largely recognized that SSc development and manifestations are the result of three different processes: (i) immune system activation/dysregulation, resulting in the production of autoantibodies, (ii) widespread peripheral microvasculopathy, and (iii) advanced interstitial and perivascular fibrosis of the skin and multiple internal organs [1,2,3,4]. Peripheral microvascular abnormalities represent the earliest pathological events of SSc, with the endothelium being one of the principal disease targets and, likely, a crucial trigger for the subsequent development of tissue fibrosis [1,2,3,4]. Fibrosis, due to an exaggerated deposition of extracellular matrix (ECM) components, such as collagen and fibronectin, alters the normal tissue architecture and often leads to significant organ dysfunction and even failure, thus gradually reducing the patient quality of life and representing one of the main causes of death [1,2,3,4,5].

In recent decades, several studies have revealed that the leading culprits of SSc-related tissue fibrosis are represented by activated myofibroblasts, a population of mesenchymal cells displaying both ECM-synthetizing properties and contractile features, which strengthen ECM stiffness, which in turn further promotes fibrosis in a kind of vicious circle [6,7,8,9]. Indeed, myofibroblasts are characterized by an excessive synthesis and secretion of type I and III collagen, a reduced production of ECM-degrading enzymes, and the expression of α-smooth muscle actin (α-SMA), a molecule that promotes contractile force generation by assembling into stress fibers [6,7,8].

On the bases of the central role exerted by myofibroblasts in the pathogenesis of organ fibrosis, growing attention has been focused on the identification of their possible origin, as their persistence and accumulation in the connective tissue undergoing remodeling cannot only be attributed to a lowered susceptibility to undergo apoptosis [8,10,11]. In this context, extensive studies have shown that profibrotic myofibroblasts may derive from different cellular sources, such as connective tissue-resident fibroblasts, the expansion and activation of microvascular pericytes, the recruitment of circulating progenitors (i.e., bone-marrow-derived fibrocytes), as well as the transdifferentiation of preadipocytes/adipocytes and epithelial cells [8,10,11]. Of note, blood vascular endothelial cells (ECs) may represent a further cellular source of myofibroblasts, as they are able to undergo the endothelial-to-myofibroblast transition (EndMT) process, a transdifferentiation during which they lose their angiogenic properties and parallelly acquire ECM-synthetizing features [7,12,13]. In particular, transitional ECs change their morphology, disaggregate, lose polarity, acquire the capacity to migrate and invade into the surrounding tissue, and undergo an immunophenotypic conversion, downregulating their typical endothelial markers, such as CD31, vascular endothelial-cadherin and von Willebrand factor, and parallelly gaining myofibroblast markers, including α-SMA; S100A4, also referred to as fibroblast-specific protein-1 (FSP1); and type I collagen [7,12,13]. During this process, ECs also exhibit stabilization and the nuclear translocation of the transcription factor Snail1, an essential inducer of the gene expression program responsible for EndMT [14,15,16].

At present, EndMT has emerged as a key contributor to the pathogenesis of several conditions, such as diabetic nephropathy, myocardial fibrosis, intestinal fibrosis complicating inflammatory bowel diseases, portal hypertension, and primary pulmonary arterial hypertension (PAH) [14,15]. Experimental evidence revealed that EndMT may also contribute to the establishment of SSc-associated interstitial lung disease and PAH [17,18], and that a variety of pathways implicated in the pathogenesis of SSc, including transforming growth factor-β (TGFβ), endothelin-1, Notch, Sonic Hedgehog, and Wnt signaling pathways, may trigger the EndMT process [15]. Furthermore, our research group has demonstrated the occurrence of a phenotypical conversion from blood vascular ECs to chronically activated/profibrotic myofibroblasts both ex vivo, in the fibrotic skin lesions of SSc patients, and in vitro, in cultures of dermal microvascular ECs explanted from clinically affected SSc skin, suggesting that EndMT may make an important contribution to the SSc-related peripheral vasculopathy characterized by microvessel rarefaction and the concomitant development of dermal fibrosis [16]. In addition, the prolonged culture with SSc serum or recombinant human TGFβ1 led healthy dermal microvascular ECs to acquire a myofibroblast-like morphology, immunophenotype, and contractile ability in culture [16].

While the role of blood microvessel dysfunction in SSc pathogenesis has been widely acknowledged, at present only a few studies have investigated the possible involvement of the lymphatic microvasculature [19,20]. Lymphatic ECs can be distinguished from blood vascular ECs by the expression of distinctive markers, such as podoplanin (PDPN), lymphatic vessel endothelial hyaluronan receptor-1 (LYVE-1), vascular endothelial growth factor receptor-3 (also known as Flt4), as well as the nuclear factor prospero-related homeobox protein 1 (Prox1), which is a main regulator of lymphatic vessel development [21,22,23]. Of note, activated profibrotic fibroblasts/myofibroblasts observed in a variety of disorders, including SSc-related cutaneous fibrosis, have been shown to express PDPN [24,25,26,27,28,29]. Furthermore, a significant reduction in the number of lymphatic microvessels correlating with the progression of tissue fibrosis has been described in skin lesions of SSc patients [19]. Finally, studies performed in mice also suggested the existence of a lymphatic endothelial-to-myofibroblast transition (Ly-EndMT) process, as testified by the fact that α-SMA expression could be induced in lymphatic ECs either in vitro by TGFβ stimulation or in vivo during wound repair, with the number of α-SMA^+^ lymphatic vessels being correlated to the severity of lymphedema and fibrosis [30]. On the basis of the aforementioned experimental data, we undertook the present study to explore the possibility that the lymphatic endothelium could represent an additional and yet-unexplored source of profibrotic myofibroblasts contributing to skin fibrosis in SSc.

## 2. Materials and Methods

### 2.1. Immunofluorescence on Human Skin Sections

To evaluate the presence of Ly-EndMT in dermal lymphatic microvessels, fluorescence immunohistochemistry was performed on archival paraffin-embedded forearm skin sections from 5 female patients with SSc classified as early diffuse cutaneous SSc (disease duration < 2 years from the first non-Raynaud’s disease symptom) and 3 age-matched female healthy donors, as described elsewhere [16]. Skin biopsies were collected following protocols approved by the institutional review board of the Azienda Ospedaliero-Universitaria Careggi (AOUC), Florence, Italy (approval number: AOUC 69/13; approval date: 17 June 2013), and the Comitato Etico Regionale per la Sperimentazione Clinica della Toscana—sezione AREA VASTA CENTRO, Florence, Italy (approval numbers: 16687_bio and 18559/bio; approval dates: 14 April 2020 and 13 April 2021). Upon antigen retrieval, autofluorescence quenching and non-specific binding site blocking, skin tissue sections (5 μm thick) were subjected to double-labeling by overnight incubation at 4 °C with antibodies against α-SMA (1:100; Cat# ab7817; Abcam, Cambridge, UK) and LYVE-1 (1:100; Cat# ab33682; Abcam) followed by a 45 min incubation at room temperature with Alexa Fluor-488-conjugated or Rhodamine Red-X-conjugated secondary antibodies (both 1:200; Invitrogen, Carlsbad, CA, USA). To identify the nuclei, slides were further stained with 4′,6-diamidino-2-phenylindole (DAPI). Negative controls obtained by omitting primary antibodies or by substituting primary antibodies with isotype- and concentration-matched irrelevant IgG were performed to confirm specificity. Fluorescent images were captured under a Leica DM4000-B microscope (Leica Microsystems, Mannheim, Germany) furnished with a Leica DFC310 FX 1.4-megapixel digital color camera and the Leica software application suite LAS V3.8 (Leica Microsystems). For each skin sample, total lymphatic vessels and lymphatic vessels displaying transitional ECs (i.e., cells coexpressing LYVE-1 and α-SMA) were counted by two observers (I.R. and M.M.) in 8 randomly chosen microscopic high-power fields (hpf; 40× original magnification), and the mean of the two independent observations constituted the final result.

### 2.2. Culture of Human Dermal Lymphatic Microvascular Endothelial Cells

Three lines of human adult dermal lymphatic microvascular ECs (HdLy-MVECs) were purchased from Lonza (HMVEC-dLyAd; Cat# CC-2810; Lonza, Basel, Switzerland). Commercial HdLy-MVECs were certified to be at least 95% double positive for CD31 and PDPN by flow cytometry. Cells were routinely cultured in EGM-2-MV complete medium (EGM-2 MV Microvascular Endothelial Cell Growth Medium-2 BulletKit; Cat# CC-3202; Lonza) at 37 °C in a 5% CO_2_ incubator. Upon reaching the confluence, HdLy-MVECs were harvested and seeded onto appropriate supports for the different experimental assays. The medium was replenished every three days, and HdLy-MVECs were used between the third and fifth culture passages.

### 2.3. Collection of Serum Samples

Serum samples were obtained from six female patients fulfilling the American College of Rheumatology/European League Against Rheumatism 2013 classification criteria [31] and diagnosed with early diffuse cutaneous SSc (mean disease duration: 13.3 months; range: 6–22 months). Patients, who were not under treatment with immunosuppressive medications, corticosteroids, or other disease-modifying drugs, were recruited from the Division of Rheumatology, AOUC, Florence, Italy. The mean patient age was 39.8 years (range: 26–53 years). All patients had antinuclear antibodies, and four patients were positive for anti-topoisomerase I antibodies. As far as nailfold videocapillaroscopy findings are concerned, two patients were classified as early pattern, while the other four were classified as active pattern. Two patients presented signs of interstitial lung disease via high-resolution computer tomography of the chest. An equal number of age-matched female healthy individuals (mean age: 39.5 years; range: 27–52 years) was enrolled to obtain control serum samples. Briefly, fresh venous blood samples were left to clot for 30 min and centrifuged at 1500× *g* for 15 min, followed by serum collection and storage in aliquots in an ultra-low temperature refrigerator at −80 °C until used for the in vitro assays. The study protocol followed the principles of the Declaration of Helsinki and was approved by the local institutional review board at the AOUC (approval number: AOUC 69/13; approval date: 17 June 2013) and the Comitato Etico Regionale per la Sperimentazione Clinica della Toscana—sezione AREA VASTA CENTRO (approval number: 18559/bio; approval date: 13 April 2021). Informed consent was obtained from all individuals involved in the study.

### 2.4. Cell Stimulation and Analysis of Cell Morphology

For cell stimulation, HdLy-MVECs were grown to 70% confluence, washed three times with serum-free medium, and subsequently serum-starved overnight in EBM-2 basal medium (Cat# CC-3156; Lonza) supplemented with 2% fetal bovine serum. The following day, the culture medium was removed, and the cells were challenged with recombinant human TGFβ1 (10 ng/mL; PeproTech, Rocky Hill, NJ, USA) or 10% serum from patients with SSc (*n* = 6) and healthy donors (*n* = 6) for 48 or 72 h. Each serum sample was examined individually and replenished every day. Cells were assayed for gene expression upon 48 h stimulation, while protein expression and contractile ability were analyzed after 72 h of stimulation. To assess cell morphology, phase-contrast microphotographs were acquired under a Leica inverted microscope (Leica Microsystems), while Alexa 488-labeled phalloidin (1:40; Invitrogen) was employed in order to visualize the F-actin cytoskeleton arrangement. The nuclei were counterstained with DAPI, and fluorescent images were acquired under a Leica DM4000-B microscope (Leica Microsystems). Elongated spindle-shaped cells, as well as cells with stress fibers, were counted in 6 randomly chosen microscopic hpf (40× original magnification) per sample by two independent observers (E.R. and M.M.) who were blinded about the sample classification. For each sample, values of the two independent counts were averaged to obtain the final result.

### 2.5. RNA Isolation, cDNA Synthesis and Quantitative SYBR Green Real-Time PCR

Forty-eight h after stimulations, HdLy-MVECs were collected, and the total RNA was purified using the Qiagen RNeasy Micro Kit (Qiagen, Milan, Italy). The synthesis of first strand cDNA was carried out using the QuantiTect Reverse Transcription kit (Qiagen). To quantify mRNA levels in the different samples, SYBR Green real-time PCR with melting curve analysis was executed in a 96-well StepOnePlus Real-Time PCR System (Applied Biosystems, Milan, Italy). For each gene, a QuantiTect Primer Assay featuring a predesigned oligonucleotide primer pair was purchased from Qiagen, as detailed in Table 1. The PCR mixture was constituted by 1 μL cDNA, sense and antisense primers (0.5 μM each), 10 μL 2× QuantiTect SYBR Green PCR Master Mix with SYBR Green I dye, ROX passive reference dye, HotStarTaq DNA Polymerase, dNTP mix, and MgCl_2_ (all from Qiagen). A standard amplification protocol was performed according to the manufacturer instructions. Dissociation curve analysis and samples with the omission of the template or the enzyme in the reverse transcription step were executed to exclude non-specific signals due to primer dimers or contamination by genomic DNA. In each sample, 18S ribosomal RNA (assay ID: Hs_RRN18S_1_SG; Cat# QT00199367; Qiagen) was assessed as an endogenous control for the normalization of the amounts of loaded cDNA. Threshold cycle (Ct) and the comparative Ct method for relative quantification were employed to calculate differences in gene expression among samples. All measurements were performed in triplicate.

### 2.6. Western Blotting

Whole protein lysates of HdLy-MVECs were prepared after 72 h upon cell stimulations, and the protein amount of each sample was assayed using the Micro BCA Protein Assay Kit (Cat# 23235; Thermo Fisher Scientific, Waltham, MA, USA). Protein lysates (40 µg of total proteins each) were subjected to the addition of Laemmli sample buffer (Bio-Rad, Hercules, CA, USA) and β-mercaptoethanol before being boiled at 100 °C for 5 min. The samples were then electrophoresed on precast polyacrylamide gels (4–15% Mini-Protean TGX Gels; Bio-Rad) followed by blotting onto nitrocellulose membranes (Bio-Rad). The membranes were subsequently incubated for 30 min at room temperature on a rotary shaker with blocking solution (WesternBreeze Chromogenic Western Blot Immunodetection Kit; Cat# WB7105; Invitrogen), and then for 1 h at room temperature with the following primary antibodies: rabbit monoclonal anti-Prox1 (1:1000; Cat# ab199359; Abcam), rabbit monoclonal anti-LYVE-1 (1:1000; Cat# ab183501; Abcam), mouse monoclonal anti-PDPN (1:200; Cat# ab10288; Abcam), rabbit monoclonal anti-S100A4 (1:1000; Cat# ab124805; Abcam), mouse monoclonal anti-α-SMA (1:300; Cat# ab7817; Abcam), rabbit monoclonal anti-COL1A1 (1:1000; Cat# 39952; Cell Signaling Technology, Danvers, MA, USA), rabbit polyclonal anti-Smad3 (1:1000; Cat# 9513S; Cell Signaling Technology), rabbit monoclonal anti-phosphorylated-Smad3 (Ser423/425) (1:1000; Cat# 9520S; Cell Signaling Technology), rabbit polyclonal anti-α-tubulin (1:1000; Cat# 2144; Cell Signaling Technology), and mouse monoclonal anti-glyceraldehyde 3-phosphate dehydrogenase (GAPDH) (1:5000; Cat# ab8245; Abcam). Either α-tubulin or GAPDH were used as control invariant proteins for normalization. Immunodetection was executed according to the instructions of the WesternBreeze Chromogenic Western Blot Immunodetection Kit (Invitrogen). The intensity of the bands was quantified using ImageJ software, version 1.53t (NIH, Bethesda, MD, USA; online at http://rsbweb.nih.gov/ij, accessed on 15 February 2023), and each protein value was normalized to the respective α-tubulin or GAPDH value, as appropriate.

### 2.7. Fluorescence Immunocytochemistry

HdLy-MVECs were seeded onto glass coverslips and stimulated as described above for 72 h. Cells were subsequently fixed with 3.7% buffered paraformaldehyde, permeabilized with a solution of 0.1% Triton X-100 in phosphate-buffered saline, and washed three times with phosphate-buffered saline at room temperature. Slides were then incubated with a blocking solution containing 1% bovine serum albumin in phosphate-buffered saline for 1 h at room temperature, and finally exposed overnight at 4 °C to the following primary antibodies: anti-Prox1 (1:200; Cat# ab199359; Abcam), anti-LYVE-1 (1:100; Cat# ab33682; Abcam), anti-S100A4 (1:100; Cat# ab124805; Abcam), anti-α-SMA (1:100; Cat# ab7817; Abcam), and anti-Snail1 (1:50; Cat# ab167609; Abcam). On the following day, cell specimens were incubated for 45 min at room temperature in the dark with either Alexa Fluor-488-conjugated or Rhodamine Red-X-conjugated IgG as secondary antibodies (both 1:200; Invitrogen). Negative controls were obtained by the incubation of the specimens with irrelevant isotype- and concentration-matched mouse or rabbit IgG (Sigma-Aldrich, St. Louis, MO, USA), as needed. The nuclei were stained blue with DAPI. Immunolabeled cells were examined using a Leica DM4000-B microscope (Leica Microsystems), and fluorescence microphotographs were acquired using a Leica DFC310 FX 1.4-megapixel digital color camera equipped with LAS V3.8 software (Leica Microsystems). Cells displaying immunopositivity for S100A4 and α-SMA, as well as Prox1^+^ and Snail1^+^ nuclei, were counted in 6 randomly chosen microscopic hpf (40× original magnification) per sample by two independent observers (I.R. and M.M.) who were blinded about the sample classification. The values of the two independent counts were averaged to obtain the final result for each sample.

### 2.8. Collagen Gel Contraction Assay

A collagen gel contraction assay was performed using a floating matrix model (CytoSelect 24-Well Cell Contraction Assay Kit; Cat# CBA-5020; Cell Biolabs, San Diego, CA, USA) following the instructions provided by the manufacturer. Briefly, HdLy-MVECs, treated as previously described for 72 h before the assay, were harvested, pelleted, and re-suspended in serum-free medium at a concentration of 5 × 10^6^ cells/mL. For each experimental point, the cell suspension (100 µL) was mixed with cold neutralized collagen gel solution (400 µL) and subsequently pipetted into one well of the adhesion resistant matrix-coated 24-well plate. Collagen gels with embedded cells were allowed to polymerize for 1 h at 37 °C in a humidified incubator with 5% CO_2_. After gel polymerization, 1 mL of basal medium or medium containing the different stimuli (i.e., SSc serum, healthy serum, and recombinant human TGFβ1) was added to the top of each collagen gel. After 24 h, the entire culture dish was scanned, and the area of each collagen gel lattice was quantified using ImageJ software, version 1.53t (NIH; online at http://rsbweb.nih.gov/ij, accessed on 5 April 2023). Negative controls consisted of collagen gels without cells. All experimental points were performed in triplicate.

### 2.9. TGFβ1 Enzyme-Linked Immunosorbent Assay

Serum levels of TGFβ1 were assessed by commercial enzyme-linked immunosorbent assay according to the manufacturer’s instructions (TGF-beta 1 Quantikine ELISA kit; Cat# DB100C; R&D Systems, Minneapolis, MN, USA). The assay sensitivity was 5.5 pg/mL. Each sample was measured in triplicate.

### 2.10. Statistical Analysis

The statistical analysis was performed using Statistical Package for Social Sciences (SPSS) software for Windows, version 28.0 (SPSS, Chicago, IL, USA). All data are expressed as mean ± standard error of the mean (SEM). The normality of the data distribution was assessed using the Kolmogorov–Smirnov test. One-way ANOVA with post hoc Tukey’s test or unpaired Student’s *t*-test were used for statistical analysis of the different sets of data, as appropriate. A non-parametric Mann–Whitney U test was performed to analyze the enzyme-linked immunosorbent assay data. Values of *p* < 0.05 were considered statistically significant.

## 3. Results

### 3.1. Detection of Ly-EndMT in the Lesional Skin of Patients with SSc

To explore the possible presence of transitional Ly-EndMT cells, forearm skin sections obtained from the clinically affected skin of patients with SSc and from healthy donors were subjected to double fluorescence immunohistochemistry for the lymphatic EC-specific marker LYVE-1 in combination with α-SMA as a myofibroblast marker.

**Figure 1:**
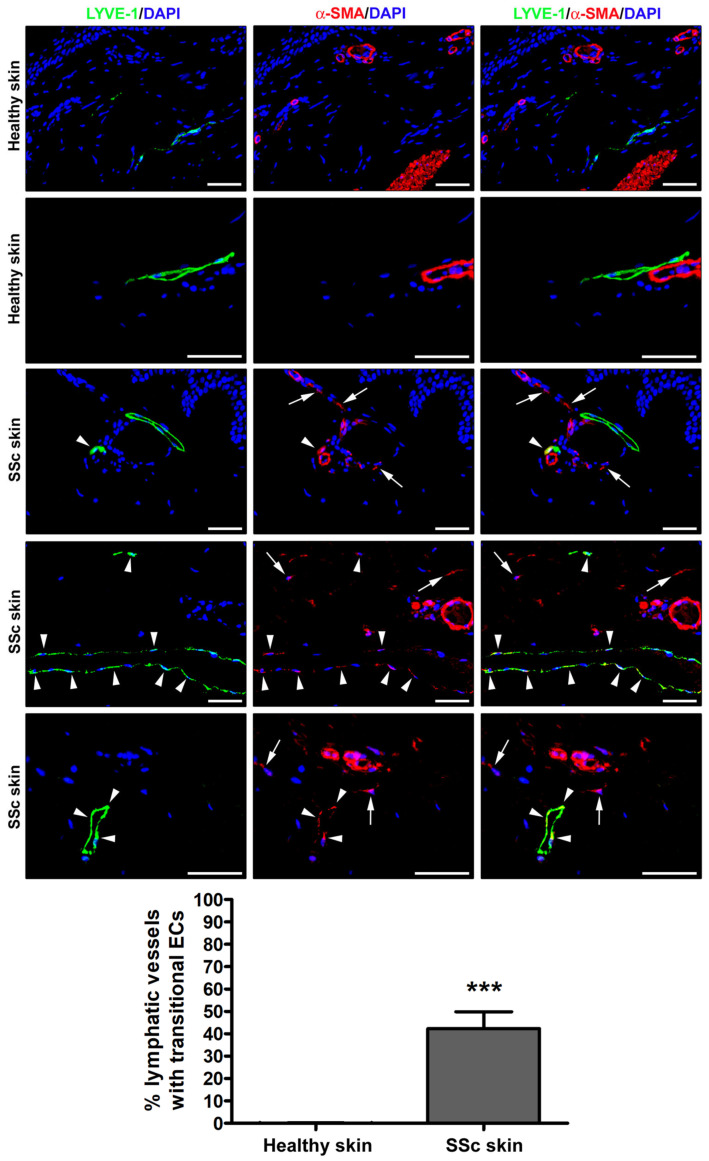
Presence of lymphatic endothelial-to-myofibroblast transition (Ly-EndMT) in dermal lymphatic capillaries of SSc patients. Representative fluorescence microphotographs of skin sections from healthy donors (*n* = 3) and patients with SSc (*n* = 5) double immunostained for the lymphatic endothelial cell-specific marker LYVE-1 (green) and the myofibroblast marker α-SMA (red). The nuclei are stained blue with 4′,6-diamidino-2-phenylindole (DAPI). In healthy skin, the expression of α-SMA is detectable exclusively in the pericytes and vascular smooth muscle cells of dermal blood microvessels and in the arrector pili muscles. In the fibrotic skin of SSc patients, colocalized LYVE-1 and α-SMA is evident as yellow staining in the transitional Ly-EndMT cells of dermal lymphatic capillaries (arrowheads). Arrows point to α-SMA^+^ stromal myofibroblasts detected in SSc skin. Scale bar = 50 μm. Bars represent the mean ± SEM of the percentage of dermal lymphatic vessels displaying transitional endothelial cells per high-power field (40× original magnification). Unpaired Student’s *t*-test was used for statistical analysis. *** *p* < 0.001 vs. healthy skin. LYVE-1: lymphatic vessel endothelial hyaluronan receptor-1; α-SMA: α-smooth muscle actin; SSc: systemic sclerosis; ECs, endothelial cells.

As shown in Figure 1, in the healthy dermis, the expression of α-SMA was detectable only in the pericytes and vascular smooth muscle cells of blood microvessels and in the arrector pili muscles. Conversely, the endothelial layer of approximately 40% of dermal lymphatic capillaries from SSc skin sections featured cells with a clear colocalization of LYVE-1 and α-SMA, thus indicating the presence of cells in the intermediate stages of the Ly-EndMT process (Figure 1). As expected, stromal myofibroblasts displaying α-SMA immunopositivity were found exclusively in the fibrotic skin of SSc patients (Figure 1).

### 3.2. Treatment with SSc Serum Induces the Acquisition of a Myofibroblast-Like Profibrotic Phenotype in HdLy-MVECs

To assess whether the SSc pathologic microenvironment was able to induce the Ly-EndMT process, three cell lines of normal HdLy-MVECs from different donors were challenged with serum from patients with SSc and healthy subjects and successively analyzed for changes in cell morphology, as well as in the gene and protein expression levels of lymphatic endothelial cell markers and myofibroblast markers. Treatment with recombinant human TGFβ1 was executed in parallel experimental points and used as positive control of Ly-EndMT [32,33,34].

**Figure 2:**
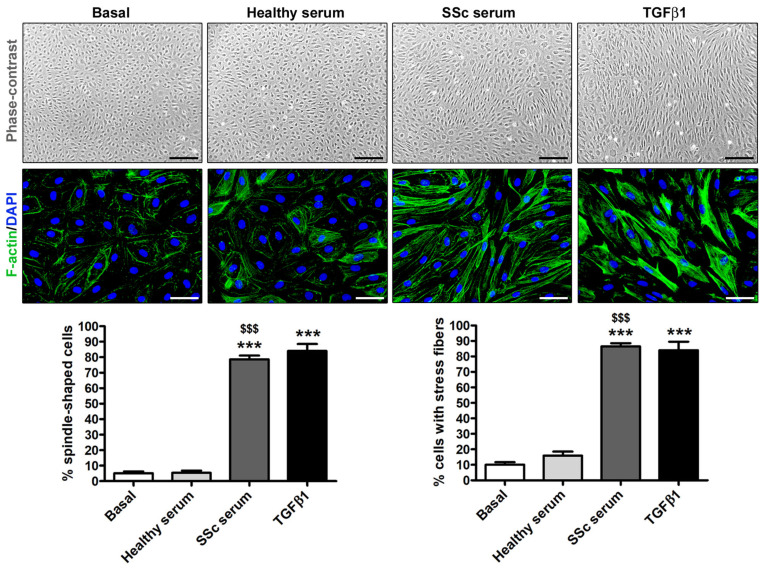
Treatment with serum from SSc patients leads to the acquisition of myofibroblast-like morphological features in healthy dermal lymphatic microvascular endothelial cells (HdLy-MVECs). The representative phase-contrast microphotographs of HdLy-MVECs (*n* = 3 cell lines) at basal condition and after 72 h culture with serum from healthy controls (*n* = 6), serum from SSc patients (*n* = 6), or recombinant human TGFβ1 are shown in the upper panels. Scale bar = 400 μm. The representative fluorescence microphotographs of HdLy-MVECs at the same experimental conditions and stained for filamentous actin (F-actin) with Alexa 488-conjugated phalloidin (green) are shown in the middle panels. The nuclei are stained blue with 4′,6-diamidino-2-phenylindole (DAPI). Scale bar = 50 μm. When compared to the basal condition, HdLy-MVEC morphology does not change in cultures challenged with serum from healthy donors. Upon treatment with SSc serum or TGFβ1, HdLy-MVECs lose their characteristic polygonal cobblestone-like morphology and exhibit an elongated spindle-shaped morphology. Bars in the lower panels represent the mean ± SEM of the percentage of cells with an elongated spindle-shaped morphology (**left**) or cells with stress fibers (**right**) per high-power field (40× original magnification). One-way ANOVA with post hoc Tukey’s test was used for statistical analysis. *** *p* < 0.001 vs. basal condition; $$$ *p* < 0.001 vs. healthy serum. TGFβ1: transforming growth factor-β1; SSc: systemic sclerosis.

As displayed in Figure 2, the culturing for 72 h with SSc serum caused HdLy-MVECs to lose their distinctive polygonal cobblestone-like EC morphology and to acquire a fibroblast-like elongated spindle-shaped appearance. Similar findings were obtained when HdLy-MVECs were stimulated with TGFβ1, while HdLy-MVECs morphological features did not change upon treatment with serum from healthy donors (Figure 2).

The remarkable cytoskeletal rearrangement triggered in HdLy-MVECs by culturing with SSc serum or TGFβ1 is shown in the lower panels of Figure 2. Indeed, while HdLy-MVECs at basal condition or after treatment with healthy serum presented mainly with a cortical actin cytoskeleton organization (i.e., actin fibers arranged at the cell periphery), those challenged with SSc serum or TGFβ1 underwent a profound rearrangement of their cytoskeleton, with actin assembling into longitudinal stress fibers, which are characteristic of myofibroblasts (Figure 2).

**Figure 3:**
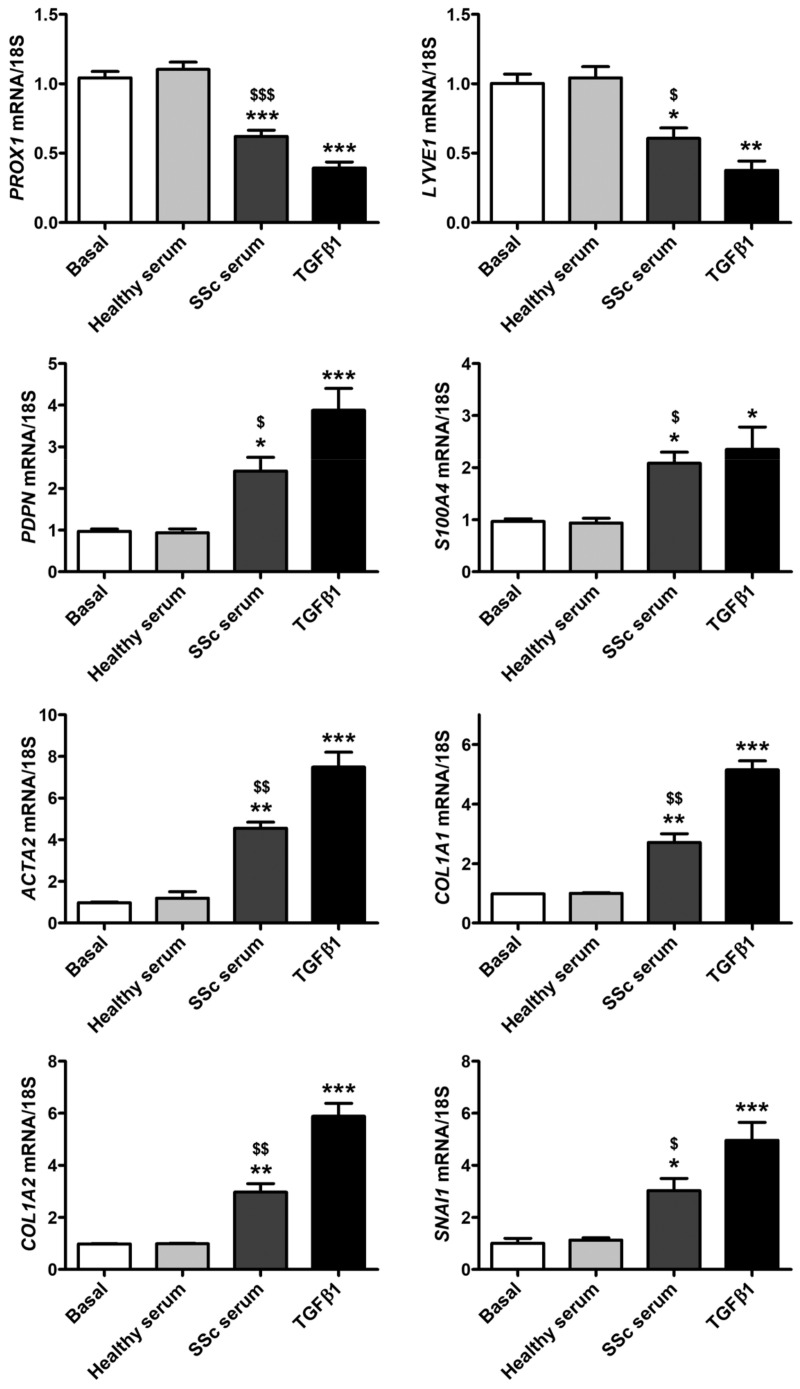
Culture with serum from SSc patients induces changes in gene expression levels of lymphatic endothelial cell-specific markers and myofibroblast markers in healthy dermal lymphatic microvascular endothelial cells (HdLy-MVECs). HdLy-MVECs were subjected to 48 h treatment with serum from healthy controls (*n* = 6), serum from SSc patients (*n* = 6), or recombinant human TGFβ1, and then examined for the expression levels of *PROX1*, *LYVE1*, *PDPN*, *S100A4*, *ACTA2*, *COL1A1*, *COL1A2*, and *SNAI1* genes by quantitative SYBR Green real-time PCR. For each gene, the basal expression level was set to 1 for the normalization of the other results. As a reference gene, 18S ribosomal RNA was used in all real-time PCR assays. Bars represent the mean ± SEM of triplicate gene expression determinations from three cell lines. One-way ANOVA with post hoc Tukey’s test was used for statistical analysis. * *p* < 0.05, ** *p* < 0.01, *** *p* < 0.001 vs. basal condition; $ *p* < 0.05, $$ *p* < 0.01, $$$ *p* < 0.001 vs. healthy serum. TGFβ1: transforming growth factor-β1; SSc: systemic sclerosis.

As shown in Figure 3, quantitative real-time PCR revealed a significant decrease in the mRNA levels of *PROX1* and *LYVE1* lymphatic endothelial-specific genes in HdLy-MVECs treated for 48 h with serum from SSc patients or TGFβ1. This was paralleled by a significant induction of the gene expression of *PDPN* (i.e., gene coding for podoplanin), *S100A4*, *ACTA2* (i.e., gene coding for α-SMA), *COL1A1* (i.e., gene coding for α-1 chain of type I collagen), *COL1A2* (i.e., gene coding for α-2 chain of type I collagen), and *SNAI1* (i.e., gene coding for the transcription factor Snail1). Conversely, the treatment with serum from healthy individuals did not change the expression levels of either lymphatic endothelial or myofibroblast genes (Figure 3).

**Figure 4:**
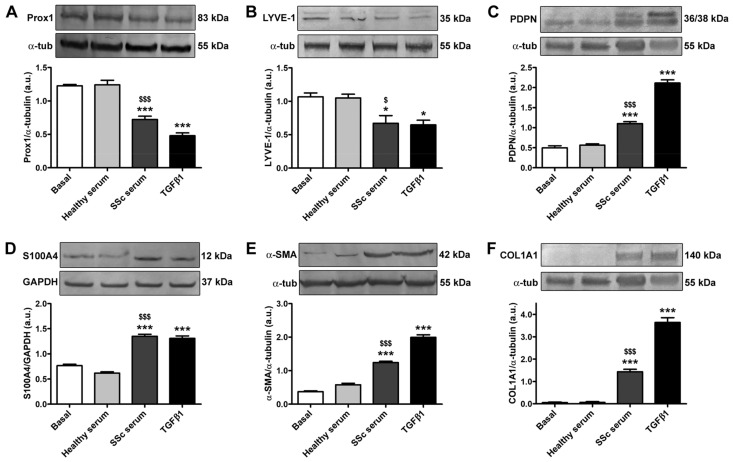
Culture with SSc serum induces changes in protein expression of lymphatic endothelial cell markers and myofibroblast markers in healthy dermal microvascular endothelial cells (HdLy-MVECs). HdLy-MVECs were subjected to 72 h challenge with serum from healthy controls (*n* = 6), serum from patients with SSc (*n* = 6), or recombinant human TGFβ1, and then analyzed by Western blotting for the protein expression of Prox1 (**A**), LYVE-1 (**B**), PDPN (**C**), S100A4 (**D**), α-SMA (**E**), and COL1A1 (**F**). Representative immunoblots are shown. The molecular weight in kDa is reported on the right of the bands. α-tubulin and GAPDH were used for normalization (loading controls). Bars represent the mean ± SEM of optical density in arbitrary units (a.u.). One-way ANOVA with post hoc Tukey’s test was used for statistical analysis. * *p* < 0.05, *** *p* < 0.001 vs. basal condition; $ *p* < 0.05, $$$ *p* < 0.001 vs. healthy serum. Prox1: prospero-related homeobox protein 1; LYVE-1: lymphatic vessel endothelial hyaluronan receptor-1; PDPN: podoplanin; α-SMA: α-smooth muscle actin; COL1A1: α-1 chain of type I collagen; TGFβ1: transforming growth factor-β1; SSc: systemic sclerosis.

Similar experimental data were obtained by Western blotting when evaluating the protein expression of lymphatic endothelial cell markers and myofibroblast markers after 72 h of HdLy-MVEC stimulation in culture (Figure 4). In particular, cells at basal conditions or stimulated with serum from healthy donors exhibited the expression of Prox1, LYVE-1 and PDPN; very low levels of S100A4 and α-SMA; and a negligible expression of COL1A1 (Figure 4). The culture of HdLy-MVECs with SSc serum or TGFβ1 resulted in a significant reduction in the protein levels of Prox1 and LYVE-1, and a parallel increase in those of the aforementioned myofibroblast markers, as well as of PDPN (Figure 4).

**Figure 5:**
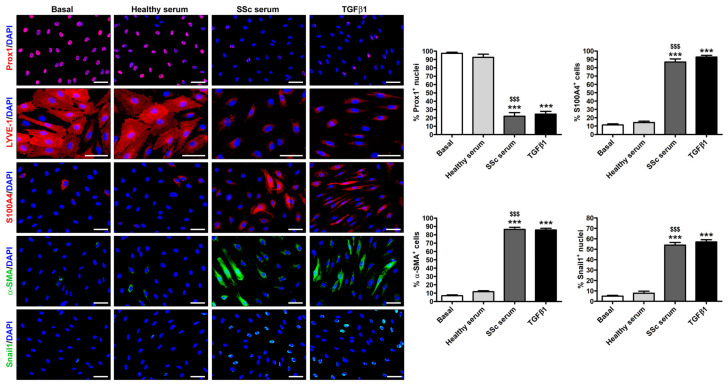
Culture with SSc serum induces a myofibroblast-like immunophenotype in healthy dermal lymphatic microvascular endothelial cells (HdLy-MVECs). Representative fluorescence microphotographs of HdLy-MVECs at basal condition and after treatment for 72 h with healthy serum (*n* = 6), SSc serum (*n* = 6), or recombinant human TGFβ1 immunostained for Prox1, LYVE-1, S100A4, α-SMA, and Snail1. The nuclei are stained blue with 4′,6-diamidino-2-phenylindole (DAPI). In HdLy-MVECs at basal condition and cultured in the presence of healthy serum, the expression of α-SMA, S100A4, and Snail1 is negligible. HdLy-MVECs challenged with SSc serum or TGFβ1 are characterized by the strong downregulation of nuclear Prox1 and cell surface LYVE-1, and by the parallel upregulation of α-SMA, partly assembled into stress fibers, as well as S100A4 and nuclear Snail1. Scale bar = 50 μm. Bars represent the mean ± SEM of the percentage of immunopositive cells or nuclei per high-power field (40× original magnification). One-way ANOVA with post hoc Tukey’s test was used for statistical analysis. *** *p* < 0.001 vs. basal condition; $$$ *p* < 0.001 vs. healthy serum. Prox1: prospero-related homeobox protein 1; LYVE-1: lymphatic vessel endothelial hyaluronan receptor-1; α-SMA: α-smooth muscle actin; TGFβ1: transforming growth factor-β1; SSc: systemic sclerosis.

The downregulation of the lymphatic endothelial nuclear factor Prox1 and of the cell surface receptor LYVE-1, along with a parallel increase in the myofibroblast markers S100A4, α-SMA, and Snail1 upon treatment of HdLy-MVECs with SSc serum or TGFβ1 was also confirmed by immunofluorescence analysis (Figure 5). Notably, both SSc serum and TGFβ1 resulted in the presence of α-SMA^+^ stress fibers and the robust nuclear localization of the transcription factor Snail1 (Figure 5). In particular, semiquantitative analysis revealed that the percentages of S100A4^+^ and α-SMA^+^ cells/hpf, as well as that of Snail1^+^ nuclei/hpf, were significantly increased in HdLy-MVECs cultured with SSc serum or TGFβ1 compared to those maintained in basal medium or treated with healthy serum (Figure 5). Under the same experimental conditions, the percentage of Prox1^+^ nuclei/hpf was significantly decreased (Figure 5).

**Figure 6:**
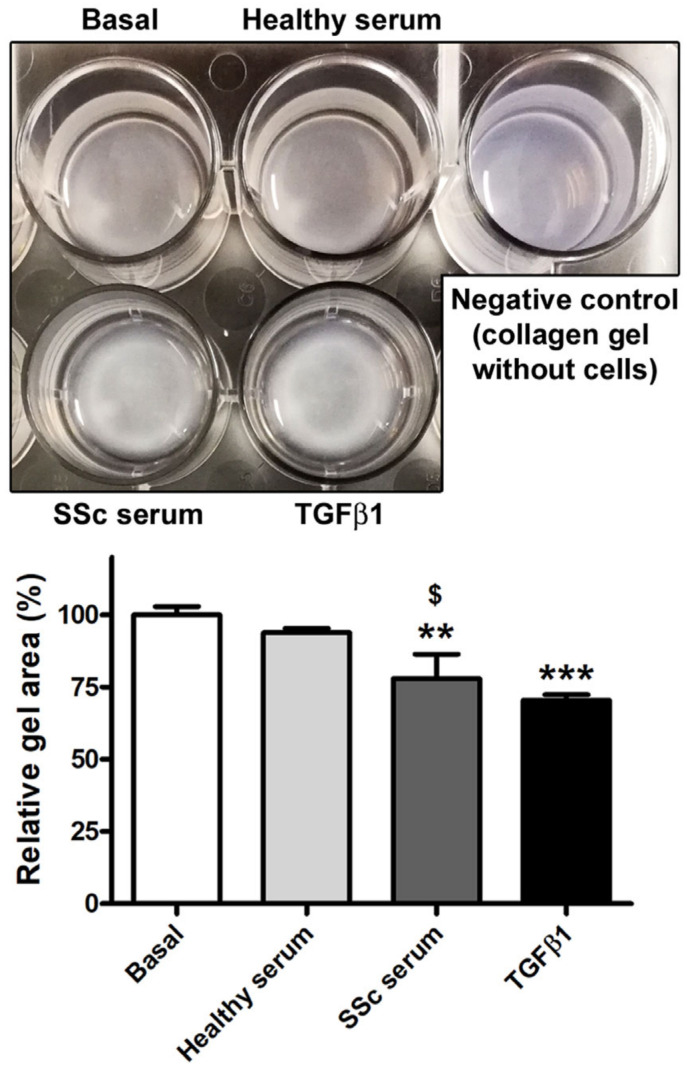
Healthy dermal lymphatic microvascular endothelial cells (HdLy-MVECs) acquire a myofibroblast-like functional phenotype after culture with serum from SSc patients. The upper panel shows representative pictures of collagen gels with HdLy-MVECs at basal condition and after treatment for 72 h with serum from healthy donors (*n* = 6), serum from SSc patients (*n* = 6), or recombinant human TGFβ1. Gel size in the presence of unstimulated HdLy-MVECs (basal) was assumed to be 100% for the normalization of the other results. Negative control consisted of collagen gel without embedded cells. Bars represent the mean ± SEM of triplicate determinations from three cell lines. One-way ANOVA with post hoc Tukey’s test was used for statistical analysis. ** *p* < 0.01, *** *p* < 0.001 vs. basal condition; $ *p* < 0.05 vs. healthy serum. TGFβ1: transforming growth factor-β1; SSc: systemic sclerosis.

To further investigate the ability of SSc serum to trigger the Ly-EndMT process, HdLy-MVECs stimulated for 72 h with serum from healthy donors, serum from SSc patients, or TGFβ1 (positive control) were also characterized from a functional point of view by assessing their contractile properties using the collagen gel contraction assay. Unlike basal HdLy-MVECs or those cultured in the presence of healthy serum, HdLy-MVECs challenged with SSc serum or TGFβ1 gained a myofibroblast-like functional phenotype and were able to significantly contract the collagen gels (Figure 6).

**Figure 7:**
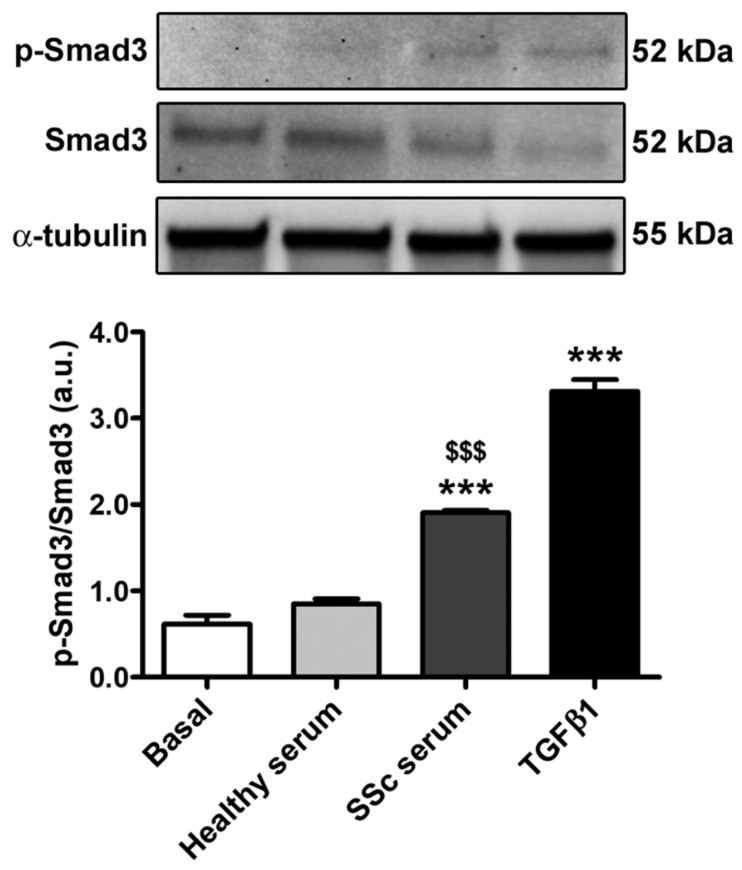
Culture with SSc serum activates Smad-dependent TGFβ signaling in healthy dermal lymphatic microvascular endothelial cells (HdLy-MVECs). HdLy-MVECs were subjected to 72 h challenge with serum from healthy controls (*n* = 6), serum from patients with SSc (*n* = 6), or recombinant human TGFβ1, and then analyzed by Western blotting for the protein expression of p-Smad3, Smad3 and α-tubulin (loading control). Representative immunoblots are shown. Molecular weight values (kDa) are indicated on the right of the bands. Bars represent the mean ± SEM of optical density in arbitrary units (a.u.). One-way ANOVA with post hoc Tukey’s test was used for statistical analysis. *** *p* < 0.001 vs. basal condition; $$$ *p* < 0.001 vs. healthy serum. p-Smad3: phosphorylated-Smad3; TGFβ1: transforming growth factor-β1; SSc: systemic sclerosis.

Finally, we investigated whether treatment with SSc serum could activate Smad-dependent canonical TGFβ signaling in HdLy-MVECs. We first measured the levels of TGFβ1 by enzyme-linked immunosorbent assay in the SSc serum samples that were used to stimulate HdLy-MVECs in the in vitro assays, and found that they were significantly increased (mean ± SEM, 91.39 ± 17.32 pg/mL) compared to serum samples from healthy donors (mean ± SEM, 36.06 ± 10.28 pg/mL; *p* = 0.02). Indeed, the culture of HdLy-MVECs with SSc serum or recombinant human TGFβ1 resulted in a significant increase in the levels of phosphorylated-Smad3 compared to both cells treated with healthy serum and those cultured in basal medium (Figure 7).

## 4. Discussion

The present study is the first to suggest the possible occurrence of a Ly-EndMT process in the clinically affected skin of patients with SSc, with the lymphatic endothelium representing an additional source of α-SMA^+^ profibrotic myofibroblasts that can contribute to the pathogenesis of disease-related cutaneous fibrosis. Indeed, our ex vivo fluorescence immunohistological findings show the presence of lymphatic ECs in intermediate stages of Ly-EndMT in SSc dermis, as testified by the coexpression of LYVE-1 and α-SMA in the endothelium bordering the lumen of lymphatic microvessels. In addition, our in vitro results indicate that treatment with serum from SSc patients is able to induce Ly-EndMT in HdLy-MVECs in a similar extent to TGFβ1, thus further supporting the notion that this mesenchymal transdifferentiation process may be effective in SSc. Indeed, the culture with SSc serum led HdLy-MVECs to lose their characteristic endothelial cobblestone-like appearance and to acquire myofibroblast-like morphological and functional features. Together with such morphofunctional changes, HdLy-MVECs challenged with SSc serum also exhibited a reduction in the expression of the lymphatic markers Prox1 and LYVE-1 accompanied by a robust increase in the myofibroblast markers S100A4, α-SMA, COL1A1, and nuclear Snail1. In agreement with our data, lymphatic ECs were found to exhibit a profibrotic transcriptome profile featuring a significant upregulation of the myofibroblast marker S100A4 in a very recent single-cell analysis of SSc skin biopsies [35].

It is well established that lymphatic ECs are defined by the expression of typical markers, such as Prox1, LYVE-1, Flt4, and PDPN [21,22,23]. However, it is important to consider that even profibrotic α-SMA^+^ fibroblasts have been demonstrated to express PDPN in a variety of disorders, including SSc-related cutaneous fibrosis and cardiac fibrosis [25,27], and that PDPN can be considered not only a reliable myofibroblast marker [24], but also a marker of aggressiveness in tumors, as this transmembrane glycoprotein promotes cancer cell migration and invasion, and is highly expressed in cancer-associated fibroblasts [26,28,29]. On this basis, we indeed considered the combination of PDPN and α-SMA markers unsuitable to search for Ly-EndMT in fibrotic skin sections, and thus chose LYVE-1 as the lymphatic EC-specific marker. This was further confirmed by the subsequent in vitro experiments. In fact, we observed a decreased expression of both LYVE-1 and Prox1 in HdLy-MVECs transitioning to α-SMA^+^ myofibroblasts upon being cultured with SSc serum or TGFβl. Instead, in the same experimental conditions, PDPN showed a robust upregulation in the transdifferentiated cells.

Clinical and histological studies have reported that the skin lesions of SSc patients exhibit dermal lymphatic microcirculation abnormalities [19,20,36,37], with a significant reduction in both initial lymphatics (i.e., lymphatic capillaries) and lymphatic precollectors associated with the development of digital ulcers and the progression of dermal fibrosis [19,20]. Initial lymphatics are blind-ended and thin-walled EC tubes that present large inter-endothelial openings and a discontinuous basement membrane [38,39]. Moreover, initial lymphatic ECs possess abluminal anchoring filaments that connect them to the surrounding ECM and are essential to maintain vessel patency when tissue pressure increases, for example, during inflammation [38,39]. Because of their greater permeability, lymphatic capillaries are therefore more effective than their blood counterparts in the drainage of protein-rich fluids and in the clearance of macromolecules from the tissue interstitial space [38,39]. In fact, the disappearance of initial lymphatics accounts for an insufficient lymphatic drainage, with the consequent accumulation of blood-derived fluids and macromolecules in the interstitium. In SSc, this may lead to the characteristic early edematous disease phase, clinically manifesting with digital painless swelling (i.e., puffy fingers) and followed by the progressive development of skin fibrosis [36]. Of note, such a chronological sequence of pathologic events might be partly explained by our findings demonstrating the occurrence of Ly-EndMT, which indeed is a process consisting in lymphatic ECs progressively disappearing in order to give rise to a pool of ECM-synthesizing profibrotic myofibroblasts.

Besides accounting for lymphatic capillary network rarefaction by increasing the number of myofibroblasts, Ly-EndMT may also contribute to preventing lymphatic microcirculation recovery by impairing lymphangiogenesis, a multistep process taking place in different conditions, including wound healing, inflammation, and cancer, during which new microlymphatics are shaped through the sprouting of ECs from pre-existing lymphatic structures [40,41]. In this context, our group has recently demonstrated that the pathologic SSc microenvironment is able to deeply hamper in vitro different steps of the lymphangiogenesis process, such as HdLy-MVEC proliferation, invasion, and wound healing capacity [40]. Interestingly, defective lymphvasculogenesis also has been suggested in SSc, as demonstrated by reduced circulating levels of bone-marrow-derived lymphatic endothelial progenitor cells in patients with digital ulcers [42].

Consistent with our findings, a decrease in the lymphatic vessel network and the possible occurrence of a Ly-EndMT process have been reported in age-related diseases [34,43,44], in experimental lymphedema [30], and in vitro in lymphatic ECs infected by Kaposi’s sarcoma herpesvirus or stimulated with TGFβ [30,32,45,46,47,48,49,50]. Besides TGFβ, which is known to be the master regulator of EndMT and other mesenchymal transitions, some microRNAs (miRNAs) have been implicated in lymphatic EC transition into myofibroblasts. In particular, the overexpression of miR-9 was found to regulate key Ly-EndMT pathways [51], while miR-31-5p was shown to inhibit macrophage-dependent TGFβ production, thus preventing Ly-EndMT in mouse primary dermal lymphatic ECs [52]. Interestingly, miR-31-5p has been reported to be dysregulated in SSc, which suggests that miRNA signature might influence lymphatic EC fate in such a fibrotic disorder [53,54]. Of note, also hypoxia, a condition which is known to contribute to SSc pathogenesis [55], may induce in vitro transcriptional changes in dermal lymphatic ECs that appear to play an important role in the progression of fibrosis [56]. In fact, it has been demonstrated that in presence of 1% pO_2_, lymphatic ECs display an increased gene expression of different types of collagen and fibronectin, thus acquiring a high potential to alter the ECM composition [56].

Although our findings point out that a dysfunctional lymphatic endothelium might directly contribute to the pathogenesis of SSc-related skin fibrosis, we are aware that further in-depth studies will be required to unravel the possible molecular mechanisms underlying the Ly-EndMT process. In this context, it is reasonable to suppose that potential candidates will include a large array of mediators and signaling pathways that are known to be dysregulated in SSc and that have already been demonstrated to induce EndMT and/or Ly-EndMT, such as canonical and non-canonical TGFβ signaling, Notch, PPARγ, endothelin-1, and a variety of miRNAs [8,11]. As preliminary findings, in the present study, we observed that the induction of Ly-EndMT by SSc serum in cultures of HdLy-MVECs is accompanied by a raise in the phosphorylation of Smad3, thus suggesting the implication of canonical TGFβ signaling. Moreover, since our study demonstrated the occurrence of Ly-EndMT only ex vivo and in vitro, it will be interesting to strengthen our data through investigations using different preclinical models of SSc as well.

## 5. Conclusions

Taken together, our data suggest the possible occurrence of Ly-EndMT in the skin of SSc patients, where such a morphofunctional cell transition could potentially represent a pathogenetic link between peripheral lymphatic network dysfunction/rarefaction, which is already found in the early/edematous disease phase, and the development of dermal fibrosis. In perspective, an in-depth elucidation of the underlying molecular mechanisms might position Ly-EndMT as a novel potential therapeutic target in the fight against the cutaneous fibrogenic process.

## Figures and Tables

**Table 1:** Details of oligonucleotide primer pairs used for quantitative SYBR Green real-time PCR.

Gene Symbol	Assay ID	Cat#
*PROX1*	Hs_PROX1_1_SG	QT01006670
*LYVE1*	Hs_LYVE1_1_SG	QT00034566
*PDPN*	Hs_PDPN_1_SG	QT01015084
*S100A4*	Hs_S100A4_1_SG	QT00014259
*ACTA2*	Hs_ACTA2_1_SG	QT00088102
*COL1A1*	Hs_COL1A1_1_SG	QT00037793
*COL1A2*	Hs_COL1A2_1_SG	QT00072058
*SNAI1*	Hs_SNAI1_1_SG	QT00010010

## Data Availability

All relevant data are included within the manuscript.
